# Synthesis of Rhizobial Exopolysaccharides and Their Importance for Symbiosis with Legume Plants

**DOI:** 10.3390/genes8120360

**Published:** 2017-12-01

**Authors:** Małgorzata Marczak, Andrzej Mazur, Piotr Koper, Kamil Żebracki, Anna Skorupska

**Affiliations:** Department of Genetics and Microbiology, Maria Curie-Skłodowska University, Akademicka 19, 20-033 Lublin, Poland; mazur@hektor.umcs.lublin.pl (A.M.); piotr.koper@poczta.umcs.lublin.pl (P.K.); kamilzebracki@poczta.umcs.lublin.pl (K.Ż.); anna.skorupska@poczta.umcs.lublin.pl (A.S.)

**Keywords:** *Rhizobium*, symbiosis, exopolysaccharide, Wzx/Wzy-dependent pathway

## Abstract

Rhizobia dwell and multiply in the soil and represent a unique group of bacteria able to enter into a symbiotic interaction with plants from the Fabaceae family and fix atmospheric nitrogen inside de novo created plant organs, called nodules. One of the key determinants of the successful interaction between these bacteria and plants are exopolysaccharides, which represent species-specific homo- and heteropolymers of different carbohydrate units frequently decorated by non-carbohydrate substituents. Exopolysaccharides are typically built from repeat units assembled by the Wzx/Wzy-dependent pathway, where individual subunits are synthesized in conjunction with the lipid anchor undecaprenylphosphate (und-PP), due to the activity of glycosyltransferases. Complete oligosaccharide repeat units are transferred to the periplasmic space by the activity of the Wzx flippase, and, while still being anchored in the membrane, they are joined by the polymerase Wzy. Here we have focused on the genetic control over the process of exopolysaccharides (EPS) biosynthesis in rhizobia, with emphasis put on the recent advancements in understanding the mode of action of the key proteins operating in the pathway. A role played by exopolysaccharide in *Rhizobium*–legume symbiosis, including recent data confirming the signaling function of EPS, is also discussed.

## 1. Introduction

Rhizobia are Gram-negative soil bacteria belonging to the α- and β-proteobacteria, which can interact with legume plant hosts. Under nitrogen-limiting conditions, they induce formation of nodules on host plant roots, invade and colonize the nodules, and inside them reduce dinitrogen to ammonia, which is used by plants. Challenging environments such as soil, in which resources are scarce and living conditions fluctuant, are frequently dominated by bacterial species with large and complex genomes. Rhizobial genomes are mostly multipartite, composed of a chromosome and several plasmids. The genes related to symbiotic interaction with plants are usually located in one of the plasmids (thus called a symbiotic plasmid, pSym) or in the chromosome as symbiotic islands [1,2]. Besides the pSym, other parts of the rhizobial genome, especially non-symbiotic plasmids, can influence the symbiotic performance of bacteria by encoding additional factors such as proteins and cell surface polysaccharides that affect competitiveness and nitrogen fixation [3,4,5,6,7,8].

The *Rhizobium*–legume symbiosis is specific and depends on the exchange of signal molecules, such as flavonoids secreted by plants, which recruit compatible rhizobia [9]. Via interaction with the NodD regulatory protein, flavonoids induce the expression of rhizobial nodulation (*nod*) genes encoding enzymes needed for synthesis of specific lipo-chitooligosaccharides, called Nod factors (NF) [4,10,11,12]. NF is the key signaling factor recognized by Nod factor receptor (NFR) that initiates the first plant responses such as root hair curling where rhizobia are trapped [13,14,15,16]. Bacteria trapped as microcolonies invade and colonize the roots through tubular structures called infection threads, which are progressive ingrowths of plant cell membranes containing a matrix composed of plant cell wall material; however, EPS is unlikely to be a required structural component of the infection thread matrix [17]. Inside root hairs, bacteria proliferate and induce divisions in the root cortex cells leading to the formation of nodule primordia [17,18,19]. In the nodule primordium, rhizobia are released by endocytosis from infection threads and form compartments termed symbiosomes, where the bacteria are surrounded by host plant-derived membranes [12,20]. In symbiosomes, bacteria differentiate into bacteroids, i.e., nitrogen-fixing forms of rhizobia, which reduce dinitrogen into ammonia transferred to the host plant. In return, the bacteria are supplied with carbon and energy sources from plant photosynthesis and find a niche for growth and multiplication within legume nodules [21,22,23,24,25]. Each step of rhizobium-plant symbiosis is tightly controlled through a complex network of signaling cascades in the plant [26]. Recently, it has been demonstrated that the epidermal and cortical responses to rhizobial infection are coordinated by plant auxin and cytokinin phytohormone signaling [18,27].

Two major types of nodules are formed by legumes belonging to the Fabaceae family: indeterminate and determinate [28,29]. The indeterminate nodules formed by legumes belonging to the galegoid clade of the Papilionoideae subfamily (e.g., *Medicago*, *Trifolium*, *Vicia*, *Pisum*, and *Astragalus*), the so-called inverted repeat-lacking clade (IRLC), have persistent meristems that remain active during the whole nodule development. Inside these nodules, new generations of plant cells infected by rhizobia form a developmental gradient of morphologically distinct zones: the apical nodule meristem, invasion zone into which infection threads release rhizobia, nitrogen fixing zones, and senescent and saprophytic zones. Inside nodules of this type, bacteroids are terminally differentiated because they are irreversibly transformed to polyploidy and cannot reverse to a viable form [19,29,30,31,32]. In both types of nodules, bacteroids exhibit changes in gene expression and metabolic activity, while large changes in DNA content occur only during bacteroid differentiation in indeterminate nodules [31]. Despite bacteroid degeneration, a small fraction of rhizobial cells in the saprophytic zone of nodules remains in a viable, vegetative form, massively multiply in the infection threads, and can be recovered from the nodules after nodule senescence, thus increasing the bacterial population in the soil [33,34,35,36,37]. Terminal differentiation of bacteroids is host-controlled and dependent on the presence of nodule-specific cysteine-rich (NCR) peptides, which are similar to defensins described in galegoid species [34]. There is a suggestion that NCR peptides interfere with many aspects of bacteroid metabolism and can optimize the nitrogen-fixation process [32,38].

In the determinate nodules formed by phaseolid legumes (e.g., *Phaseolus*, *Lotus*, and *Glycine*), the meristem functions only at the beginning of nodule development, i.e., formation of nodule primordia. The nitrogen-fixing bacteroids are viable and can revert to free-living forms. Nodule growth is possible largely due to cell expansion; hence, the nodules are spherical and developmentally homogeneous [29,34,38,39,40].

## 2. Rhizobial Exopolysaccharides Play Diverse Roles in Symbiotic Interaction with Legume Plants

The development of an effective symbiosis is dependent not only on genes directly required for symbiosis and located mainly on pSym plasmids, but also on genes responsible for the production of different types of cell-surface polysaccharides [3,4]. The surface of rhizobial cells contains a variety of polysaccharides, such as outer membrane-localized lipopolysaccharide (LPS), capsule polysaccharide (CPS), gel-forming polysaccharide (GPS), exopolysaccharides (EPS), K-antigen polysaccharide (KPS), cyclic β-(1,2) glucans, and high-molecular-weight neutral polysaccharide (NP or glucomannan) [6,41,42]. LPS is anchored in the outer membrane and is composed of lipid A, a core oligosaccharide, and O-antigen polysaccharide. Lipopolysaccharide is important in later stages of symbiosis [43]. Neutral CPS form a kind of polysaccharide matrix tightly associated with the cell surface. Its insoluble gel-forming shape is synthesized in the stationary phase of growth of *Rhizobium leguminosarum* and *Rhizobium trifolii* [44]. In turn, cyclic neutral β-(1,2) glucans are located in the periplasmic space and play an important role during hypoosmotic adaptation and plant infection [45]. Rhizobial KPS structurally resemble K-antigens of *Escherichia coli* [46], while glucomannan, which is exclusively localized on one of the poles of the bacterial cell, was shown to be important for high-affinity binding of pea and vetch lectin and competitive nodulation of rhizobia [42,47].

**Figure 1:**
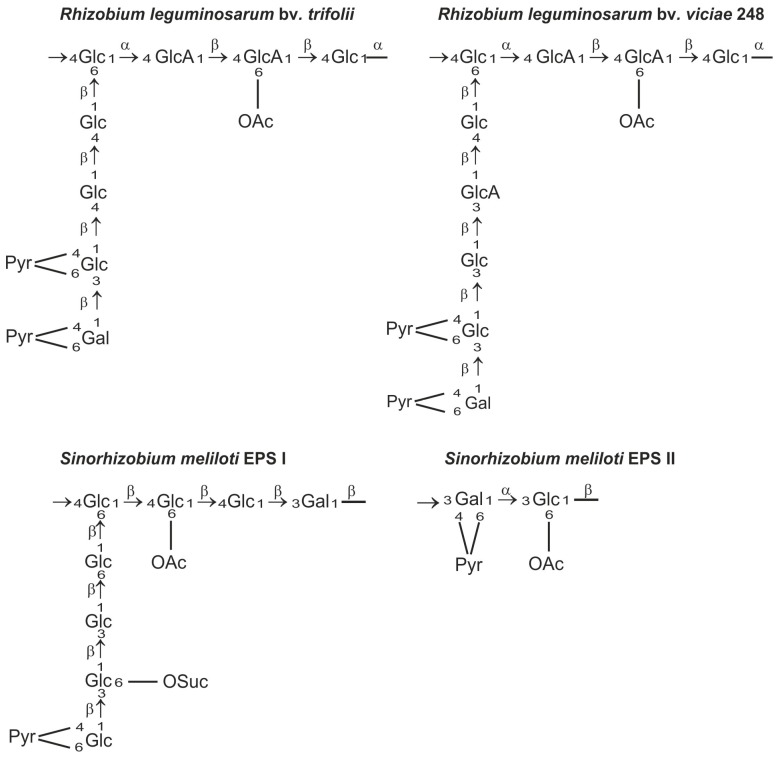
Selected chemical structures of exopolysaccharide (EPS) repeating unit of: *Rhizobium leguminosarum* bv. *trifoli* [50,51], *Rhizobium leguminosarum* bv. *viciae* [48], *Sinorhizobium meliloti* EPS I [52], *Sinorhizobium meliloti* EPS II [53]. Glc, glucose; GlcA, glucuronic acid; Gal, Galactose; OAc, acetyl; Pyr, ketal pyruvate; OSuc, succinyl groups.

Exopolysaccharides produced by rhizobia are chemically diverse species- or strain-specific heteropolymers composed of linear or branched repeating units containing monosaccharides, such as d-glucose, d-galactose, d-mannose, l-rhamnose, d-glucuronic acid, and d-galacturonic acid, usually substituted with non-carbohydrate moieties (e.g., acetyl, pyruvyl, succinyl, and 3-hydroxybutanoyl groups) [48,49,50,51] (Figure 1). The heterogeneity of exopolysaccharide structures as well as the variety of glycosidic bonds and the degree of polymerization of the repeating unit are reflected in the complex biosynthetic pathway of EPS [41].

Exopolysaccharide is an extracellular polymer that is weakly associated with the bacterial surface and thus abundantly released into the surrounding environment. The prevalence of EPS in different bacteria as well as the numerous and diverse functions attributed to this heteropolymer (including protection against stresses, biofilm formation and attachment to abiotic surfaces and host plant roots, and nutrient acquisition), which give the microbes an adaptive environmental advantage, are the reason why special attention is paid to this secreted polysaccharide [3,6].

Although there are many studies on the role of EPS in symbioses, the mechanisms in which EPSs determine beneficial host-symbiont interactions in legumes remains mostly unknown. However, several recent findings concerning the symbiotic function of EPS have been described. It shown that, in rhizobia inducing determinate nodules, EPS plays a signaling role at the late stages of both infection thread initiation and bacterial release in symbiosis with *Lotus japonicus* [54]. *Mesorhizobium loti* mutants affected in mid or late EPS biosynthetic steps induced uninfected nodule primordia and, occasionally, a few infected nodules. These mutants were disrupted at the stage of infection thread (IT) development. On the other hand, in *Sinorhizobium fredii* HH103 effectively nodulating different legumes, including plants forming determinate and indeterminate nodules, the absence of EPS increases its competitiveness to nodulate soybean, suggesting that in this interaction the EPS is dispensable but diminishes the capacity of HH103 to infect this plant [55]. In galegoid legume symbiosis, several classes of surface polysaccharides appeared to be needed for initiation and extension of infection threads and nodule development [10,43,56]. 

The role of EPS in the infection process has been most intensively studied in *Sinorhizobium meliloti* and *Rhizobium leguminosarum* symbioses. Mutants that do not produce EPS are able to induce root hair curling but not formation of infection threads and nitrogen fixing nodules [6,10,57,58,59]. *S. meliloti* produces symbiotically active succinoglycan (EPS I) and galactoglucan (EPS II). Galactoglucan is a polymer of a disaccharide repeating unit composed of an acetylated glucose and pyruvylated galactose residue [60] and it was postulated as a symbiotically important exopolysaccharide of *S. meliloti* [61]. Succinoglycan is composed of octasaccharide repeating units consisting of one galactose and seven glucose residues, and modified with succinyl, acetyl, and pyruvyl substituents. The EPS of *R. leguminosarum* is composed of octamers built of one galactose, two glucuronic acid, and five glucose residues decorated by *O*-acetyl and pyruvyl substituents (Figure 1). These EPSs are produced in two polymeric forms: low molecular weight (LMW) composed of monomers, dimers, and trimers of the basic subunit and high molecular weight (HMW) that have masses of 10^6^–10^7^ Da [6,43,62,63,64]. It has long been postulated that the LMW EPS fraction in *S. meliloti* and *R. leguminosarum* is important for efficient nodule infection [65,66,67]. However, recent data show unequivocally that *S. meliloti* mutants, which do not produce any LMW succinoglycan, establish a productive symbiosis with *Medicago truncatula*, albeit with lower efficiency than the wild type strain [68]. Previously, it was evidenced that a *S. meliloti exoH* mutant produces unsuccinylated EPS I in the form of HMW EPS, which is not cleaved by specific glycanases and induces a reduced number of inefficient nodules on alfalfa, in which the infection threads abort [69,70,71]. Mendis et al. [68] demonstrated that the double mutant deficient in ExoH and ExoK glycanases did not produce LMW EPS, but was able to establish productive symbiosis with *M. truncatula*. This indicates that the LMW EPS fraction does not play an essential role in the symbiosis; however, succinylation of EPS is required for effective nodulation. Moreover, it was evidenced that LMW hexose sugar-containing material produced by ExoH/ExoK double mutants was most likely cyclic β-glucan. These results confirmed that succinylated HMW EPS I is necessary for productive symbiosis of *S. meliloti* with the *Medicago truncatula* model plant [68]. The high molecular weight fraction of EPS can serve as a protection against desiccation of bacteria [72]. Negatively charged succinyl and pyruvyl residues in EPS I have also been shown to play important roles. The loss of succinyl groups in EPS I resulted in an increase in viscosity and in polymer chain stiffness [73]. The lack of negative charge would reduce the ability of succinoglycan to react with positively charged ions in the infection thread matrix [74]. Moreover, acylation of succinoglycan could alter its ability to quench reactive oxygen species (ROS) inside infection threads and thus affect their formation [75].

Increased amounts of acidic succinoglycan produced by *S. meliloti* overexpressing the *exoY* gene encoding a priming galactosyltransferase enhanced the symbiotic productivity of inoculated *M. truncatula*. It has been suggested that the level of exopolysaccharide produced by rhizobia can be one of the factors involved in optimizing the interaction with plant hosts [76]. In *R. leguminosarum*, the HMW EPS fraction may be beneficial at the stage of infection. It was observed that *R. leguminosarum* mutants, which produce more exopolysaccharide with a higher degree of polymerization than the wild-type strain, promote production of a higher amount of green mass of infected clover plants [63,77]. Roles that LMW and HMW EPS play in the symbiotic interaction correlate with functions that LMW and HMW polysaccharides play in other bacteria; for example, the *Shigella flexneri* O-antigen S (short chain) determines virulence of the strain [78], while the O-antigen VL (very long chain) makes the bacteria resistant to the innate immune complement system [79]. Recently, Kawaharada et al. [80] have shed light on the recognition of EPS by legume plants and the plant receptor protein interacting with rhizobial EPS was identified. *Mesorhizobium loti* produces acidic EPS composed of *O*-acetylated octamers of ribose, glucose, and galactose and enters into symbiosis with *L. japonicus* forming determined-type nodules [54,81,82,83]. A *M. loti* mutant in the *exoU* gene produced truncated EPS (pentaglycan), formed colonies with rough morphology, and induced a low number of ineffective small nodules on host plants [54]. The ExoU mutant was thus unaffected in induction of early host symbiotic responses such as root hair curling, but did not form normal infection threads. This allowed a conclusion that truncated EPS escape recognition by the mutated form of the host receptor protein thus leading to normal nodulation [80]. A suppressor mutant plant strain in gene *Epr3* designated exopolysaccharide receptor 3 was found and evidenced to form infected nodules with the *exoU* mutant. Exopolysaccharide Receptor 3 (EPR3) identified in *L. japonicus* occurred to be a membrane-spanning receptor-like protein with significant similarity to the NFR1 (Nod factor receptor 1) protein responsible for recognizing rhizobial NF, but with unique domain organization. Both EPR3 and NFR1 have three extracellular LysM domains related to chitin-binding proteins. The intracellular kinase domain present in both proteins transduces the signal from the receptor to intracellular signaling cascades. Kawaharada et al. [80] demonstrated that both wild-type EPS and a functional EPR3 receptor are required for sustained infection-thread initiation. It was evidenced in an in vitro assay that purified EPS monomers can be recognized by a purified EPR3 ectodomain by direct binding, indicating that recognition is exerted at the stage of infection and colonization of epidermal cells. *Epr3* gene expression in root hairs and epidermal cells of the susceptible zone is triggered by Nod factors signaling through NRF1 and NRF5 (serine/threonine receptor kinases), which are responsible for the first symbiotic signal transmitted to plant cells leading to several responses, including transcription of *Epr3* and perception of structurally specific EPS in infecting bacteria. There is a strong suggestion that EPR3 distinguishes between the structures of EPS and respond negatively to incompatible EPS and positively to compatible EPS [80]. In a further study, Kawaharada et al. [84] established that the action of the EPR3 receptor protein is not restricted to root hairs but advances the intracellular infection and correlates infection thread invasion of the root cortex and nodule primordia to facilitate an efficient plant infection. It was suggested that recognition of compatible EPS by EPR3 is reiterated during the progressing infection and promotes an intracellular cortical infection mechanism. The presence of EPS and EPS perception by EPR3 are important for the progression of nodule infection and affect nodule development. Mutation of *Epr3* or EPS synthesis genes reduces or eliminates nodule infection by infection threads and reduces the number of infected cells [84]. These latest results have significantly furthered our understanding of the rhizobial infection mechanism in which structurally compatible EPS can be recognized by specific host plant receptors.

## 3. Wzx/Wzy-Dependent Synthesis of Exopolysaccharide in *Rhizobium leguminosarum* bv. *trifolii*

The current state of knowledge of EPS biosynthesis was gained from comprehensive studies of succinoglycan produced by *S. meliloti* and to a lesser extent from research of EPS synthesis in a *R. leguminosarum* model, and this issue has recently been exhaustively reviewed [41,85]. Briefly, the genes directing the biosynthesis of exopolysaccharides were found to be numerous and placed in large clusters (frequently syntenic in the genomes of different rhizobial species) located either in the chromosome or in megaplasmids [86,87,88,89]. In the pSymB megaplasmid of *S. meliloti*, a large (>30 kb) cluster of 28 *exo*/*exs* genes was found. The genes code for enzymes required for the synthesis of nucleotide sugar precursors, proteins engaged in unit assembly and modification, and those required for polymerization of repeating units and transport of EPS outside the cell [90,91,92,93,94,95]. Noteworthy, several other genes essential for EPS I biosynthesis and regulation of this process were found to be dispersed throughout the chromosome of *S. meliloti* [96,97].

Generally, polysaccharides may be synthesized entirely in the cytoplasm before being transported outside the cell, or through a mechanism where repeating subunits are first synthesized in the cytoplasm, followed by transport to the periplasm for polymerization, then secretion [98,99]. Exopolysaccharides are synthesized by the second scheme referred to as Wzx/Wzy-dependent involving two key proteins: Wzx (flippase) and Wzy (polymerase). These proteins are absent in the systems of polysaccharide biosynthesis based on the activity of ATP-binding cassette (ABC)-transporters [100] or synthase (e.g., alginate produced by *Pseudomonas*) [101]. Polysaccharides such as dextran are produced by a secreted or cell-associated extracellular enzyme [99].

In the case of an ABC transporter-dependent pathway, the polysaccharide (mainly capsular) is synthesized in the cytoplasm and subsequently transported through the periplasm by an ABC -family protein. If only one glycosyltransferase is encoded within the region of the polysaccharide biosynthesis genes, produced polysaccharide is a homopolymer and if there are more genes encoding glycosyltransferases—a heteropolymer is formed [101]. Polysaccharides such as curdlan, cellulose, or alginate are synthesized and transported out of the cytoplasm by a membrane synthase, which is a processive glycosyltransferase capable of forming a polymer and promoting polymer threading through a membrane. The polymer is transported outside the cell with the participation of a periplasmic protein acting as a scaffold and preventing the polysaccharide from degradation, and a β-barrel protein located in the outer membrane. Proteins of this system are unrelated to the components of the Wzx/Wzy-dependent and ABC-transporter systems [101].

In the Wzx/Wzy-dependent pathway, oligosaccharide repeat subunits are first synthesized in conjunction with the so-called lipid anchor undecaprenylphosphate (undPP), due to the activity of specific glycosyltransferases. Biosynthetic precursors, i.e., activated nucleotide sugar derivatives (NDP-sugars), are synthesized in the cytoplasm atop und-PP. Complete oligosaccharide repeat units are transferred from the cytoplasmic to the periplasmic leaflets of the inner membrane by the activity of the Wzx flippase [102]. While still being anchored in the inner membrane, they are joined in the periplasm by the polymerase Wzy. The assembly process of the latter polysaccharide involves a protein devoid of the polymerization activity, but indispensable in the process and involved in regulation of the chain length, the so-called polysaccharide co-polymerase (PCP) [98]. The nascent polysaccharide chain is then exported outside the cell.

### 3.1. Basic EPS Subunits Are Synthesized by Glycosyltransferases

**Figure 2:**
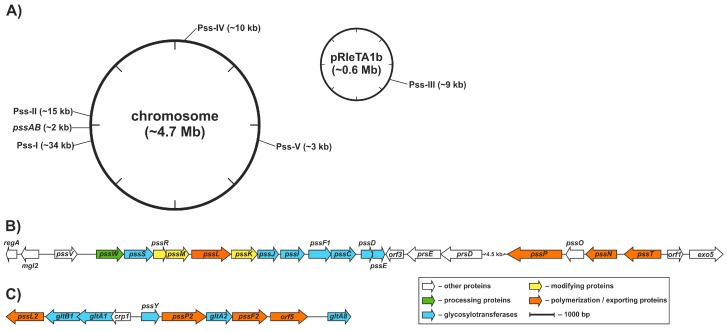
Genomic localization and genetic organization of the identified Pss regions in *Rhizobium leguminosarum* bv. *trifolii* TA1. Pss-I, Pss-II regions and *pssAB* genes are linked in the 200-kb region in the chromosome. Other Pss regions are either located distantly on the chromosome or in the pRleTA1b plasmid (**A**). Genetic organization of the Pss-I (**B**) and Pss-II (**C**) gene clusters. Pss-I region (**B**), groups majority of the experimentally characterized EPS biosynthesis-related genes. The genes located in the region Pss-II (**C**) have been identified by the similarity with the key elements of the Wzx/Wzy-dependent pathway. The genes/ORFs (Open Reading Frame) are colored in accordance with annotated or experimentally verified functions in the subsequent steps of the biosynthesis of the exopolysaccharide.

Genetic control of EPS production in *R. leguminosarum* was partially characterized at the molecular level and the functions of several genes involved in the process, *pss* genes (polysaccharide synthesis), were dissected. In *R. leguminosarum*, the core set of EPS biosynthesis genes (>20 genes) is clustered within a chromosomal region named Pss-I [87,88,89] (Figure 2). The Pss-I region contains genes encoding glycosyltransferases responsible for EPS subunit synthesis (except for *pssA* encoding a protein involved in the first step of EPS synthesis responsible for the addition of glucose-1-phosphate to a polyprenyl phosphate carrier) [103,104] and genes whose products form the EPS assembly and export system, i.e., the putative flippase [105], the polysaccharide polymerase [63], the co-polymerase protein [64], and the outer membrane channel protein [106] (Figure 2).

The Pss-I locus is highly conserved and syntenic between *R. leguminosarum* bvs. *trifolii* (*Rlt*) and *viciae* (*Rlv*) and closely related *Rhizobium etli* (*Rhe*). The preservation of a conserved gene order is usually not a random trait because an appropriate gene neighborhood confers an adaptive advantage to the cell [107]. Thus, the clustering of the main set of EPS genes seems not to be accidental and probably reflects their coordinated expression and precise, complex regulation carried at both transcriptional and post-transcriptional levels, further affected by numerous environmental factors (including light) as well as growth and stress conditions [41,108,109,110].

Numerous genes with a function in surface polysaccharide biosynthesis were also found dispersed in the *R. leguminosarum* genomes, for example an *exoB* ortholog involved in the biosynthesis of various heteropolysaccharides (EPS, CPS, LPS, GPS, glucomannan) [42,111], was found in the chromosome, regulatory genes *psi* and *psr* [112] located in symbiotic plasmid (pSym), while *rosR*, *exoR* [113,114], and *pssB* [115] genes located elsewhere in the chromosome.

There is relatively little information about glycosyltransferases, i.e., representatives of proteins involved in the biosynthesis of exopolysaccharide in rhizobia. In general, glycosyltransferases constitute a diverse family of proteins, which is reflected in the growing content of the CAZy database (Carbohydrate Active Enzymes) [116]. The relationships between the amino acid sequence of putative glycosyltransferase and its specificity is not obvious, which makes it difficult to predict its function only on the bioinformatic basis [99,117]. The functions of enzymes active in the various stages of succinoglycan subunit synthesis and phenotypic effects of mutations have been characterized in the model *S. meliloti* [94,95,118]. However, no information on the interactions between glycosyltransferases and the postulated relationships between a hypothetical glycosyltransferase complex and proteins active in the later stages of the synthesis is available. Functions of several glycosyltransferases encoded within and beyond the Pss-I region in *R. leguminosarum* bv. *trifolii* were studied previously in our group. Among them, the best studied is the priming glucosyl-isoprenylphosphate (IP)-transferase PssA, initializing octasaccharide synthesis by the transfer of glucose to lipid-bound undPP [103,119]. Glucuronosyl-β-1,4-glucosyltransferase PssDE transfers glucuronic acid to glucose [104,120], and another residue of glucuronic acid is transferred by glucuronosyl-β-1,4-glucuronosyltransferase PssC [121]. Mutations in the genes encoding glycosyltransferases can result in complete inhibition of the synthesis of EPS (*pssA*, *pssE*, *pssD*) [104,119,120] or in a decreased synthesis level, as in the case of a *pssC* mutant [122]. The latter result is interesting, as it indicates that the mutation does not block polymerization of subunits, suggesting that a specific cellular function is taken over by another protein. The enzymes engaged in the later steps of the subunit synthesis have not been investigated in detail. It is known that *pssS* encodes a hypothetical protein similar to glycosyltransferases with a GT-B fold (a class of inverting glycosyltransferases with a characteristic topology consisting of two β/α/β Rossmann domains that face each other and are linked flexibly), most probably involved in the fourth step in the unit assembly, i.e., addition of the last glucose residue to the main chain [123]. Bioinformatic analyses showed that uncharacterized hypothetical glycosyltransferase genes involved in the biosynthesis of EPS may be present among proteins encoded within the Pss-I region. It is worth noting that in all cases, data concerning glycosyltransferases in rhizobia are limited to confirmation of the participation in the biosynthesis of EPS or function defined on the basis of similarity of amino acid sequences. The mechanisms of action, substrate specificity, domain structure, or interactions between glycosyltransferases and the interactions with other proteins remain to be deciphered.

### 3.2. EPS Subunits Are Transferred Across the Inner Membrane by the Action of the Wzx Flippase

The Wzx flippase belongs to the PST (polysaccharide transporter) family of proteins in the multidrug/oligosaccharidyl-lipid/polysaccharide (MOP) exporter superfamily [124]. It represents a class of proteins whose mechanism of action has recently been elucidated. Despite the prevalence of Wzx-encoding genes in the Bacteria and Archaea domains, the level of amino acid sequence similarity between the proteins is very low. It has recently been shown that Wzx translocases are commonly adapted to their native repeat unit, which provides an explanation for the great diversity of *wzx* genes that comes along with the great diversity of structures being translocated [125]. At the same time, PST proteins are characterized by a specific topology, with 10–14 predicted TMSs (transmembrane segments) [124]. The only data concerning Wzx proteins structure are the membrane topology models for four Wzx proteins from diverse bacterial species. The common theme in all these models is the presence of 12 TMSs, even though the methodology implemented in the experiments was different [105,126,127,128]. Islam et al. [127] identified interesting (and previously omitted) features, such as the presence of charged residues within TMSs, i.e., a phenomenon that was not observed in other topological studies of flippases [127]. The Wzx specific function is to transfer the hydrophilic polysaccharide anchored on a hydrophobic carrier across the inner membrane. Recently, supporting evidence has been provided that the transfer takes place according to the mechanism of an H^+^-dependent antiport, wherein the conformational changes and rearrangements between the Wzx transmembrane segments are triggered, so that the oligosaccharide still anchored in the membrane is bound within the protein’s lumen and released on the other side of the membrane [102,127,129,130].

Components of the Wzx/Wzy-dependent assembly system, i.e., genes encoding the polymerase, co-polymerase, and translocase are usually clustered. The same is true for the Pss-I region in *R. leguminosarum* bv. *trifolii*; the exception is the flippase-encoding gene located elsewhere, however, in close proximity to the glycosyltransferase-encoding genes. The Wzx flippase in *R. leguminosarum* bv. *trifolii* is encoded by the *pssL* gene. The putative protein was first annotated as an alternative polysaccharide polymerase, because of the similarity in the topologies of these proteins in *Rhizobium* [105]. However, updated in silico analyses enabled more accurate annotation of PssL as a putative flippase, most probably acting in transferring the EPS subunits from the cytoplasmic to the periplasmic leaflet of the inner membrane. The membrane topology model of PssL was the first proposed for this family of proteins [105]. Even though the in silico obtained model with 12 TMSs was fully confirmed through a PhoA/LacZ fusion approach, some important features of the Wzx protein (e.g., the presence of positive charged amino acid residues within transmembrane segments) appeared to be overlooked because of the methodology implemented, i.e., examining the biased model made in silico [127]. The function of *pssL* in translocation of EPS subunits was not confirmed, as obtaining a null mutant in the model *R. leguminosarum* bv. *trifolii* TA1 (RtTA1) strain was not possible [105].

### 3.3. Wzy Protein Is Responsible for Polymerization of the EPS Subunits

Wzy is a polysaccharide polymerase, a protein with the activity of a glycosyltransferase linking subunits to make the polysaccharide [131]. Similar to flippases, Wzy proteins are characterized by a low level of amino acid sequence similarity and concomitantly with distinctive topology with 10–14 TMSs and 1–2 sizeable periplasmic domains (PLs; smaller at the N-terminus and greater at the C-terminus) [63,127,132]. As with the Wzx proteins, the difficulties in obtaining structural data made topology mapping important for uncovering the important functional features of these proteins. The latest topological model, described by Islam et al. [127], for the Wzy protein in *Pseudomonas aeruginosa* PAO1 was a model based on unbiased C-terminal reporter tag localization [127]. Specific amino acid motifs were identified within the large periplasmic loops PL3 and PL5 present in Wzy. These RX_10_G motifs comprise key arginine residues important for Wzy function. Despite the high degree of sequence conservation, the sequence context contributes to completely different charge characteristics of both loops, i.e., strikingly different isoelectric point (pI) values [133]. Due to the distinct net charge, these domains represent binding sites for the two versions of the same oligosaccharide i.e., a basic subunit in the form associated with the lipid carrier, then as part of the growing polysaccharide. Wzy-dependent polymerization of undPP-linked repeat units takes place in the periplasm. PL3 would function as the capture arm for the incoming negatively charged subunits. The large periplasmic loop PL5 with its more negatively charged environment would provide a site for binding and release of the growing chain. This putative mechanism was called ‘catch and release’ [133].

The importance of arginine residues was also confirmed for Wzy polymerization activity in *Shigella flexneri*. The protein has RX_15_G motif in both PL3 and PL5. However, while in Wzy in *P. aeruginosa* PAO1 Arg residues are found mainly within specific RX_10_G motifs, in *S. flexneri* Wzy there are also several Arg residues located between these two motifs. Also, the periplasmic domains of *S. flexneri* Wzy were found to possess charge characteristics different from those of Wzy in *P. aeruginosa* PAO1. The latter may be linked to differences in the net charge of oligosaccharides that are transferred by certain proteins [134]. Bioinformatic searches allowed identification of such conserved Arg residues, as well as PL3-PL5 sequence homology, in phylogenetically diverse bacteria [135]. Wzy proteins exhibit enormous sequence variation (similarly to Wzx proteins). It has recently been evidenced that these polymerases can even discriminate between the internal linkages in otherwise identical oligosaccharide units [136].

In *R. leguminosarum* bv. *trifolii*, *pssT* encoding EPS polymerase was identified before the *pssL* flippase-encoding gene. Its role in EPS synthesis was confirmed through mutagenesis. The mutant with the *pssT* gene disrupted close to the 3′-end produced more EPS than the wild type strain and the exopolysaccharide contained more chains with higher molecular weight (HMW fraction) [63]. Revealed interactions between the polysaccharide polymerase and the co polymerase (described later) in *R. leguminosarum* bv. *trifolii* and other bacteria shed light on this phenotype. Mutant in *pssT* is affected in controlling the length of the exopolysaccharide due to disturbances in protein–protein interactions within the ‘EPS polymerization center’ [77]. PssT contains 12 TMSs. At first, we postulated that the protein possesses only one large periplasmic loop (PL5). However, the recent examination of the protein sequence in search of RX_10_G motifs essential for polymerase function [133], as well as careful examination of the previously unexplainable experimental data, revealed that the topology of PssT most probably resembles that of Wzy in *P. aeruginosa* PAO1 [127] with two large PL domains and motifs containing arginine residues important for the glycosyltransferase activity of the polymerase protein [77].

### 3.4. Polysaccharide Co-Polymerases Determine the Length of Exopolysaccharide Chains

Polymerization of EPS subunits (or O-antigen repeating blocks) in the Wzx/Wzy system depends largely on other proteins devoid of glycosyltransferase activity, but indispensable in the process of polymerization. These proteins specifically influence the length of the polymer formed in a given strain and was originally classified as chain length determinants (Cld/Rol) [137] and then as membrane periplasmic auxiliary (MPA) [138] and polysaccharide co-polymerases (PCP) [139]. Recently, a group within PCP proteins has been classified as bacterial tyrosine kinases (BYK) [140,141]. Proteins belonging to this family are characterized by low conservation among different bacteria, yet concomitantly by a characteristic topology with one large periplasmic loop flanked by two transmembrane segments [138,139].

The mechanisms determining strain-specific chain lengths of polysaccharides are still not well understood. Several models have been proposed since the initial recognition of the chain-length determination phenomenon. Tocilj et al. [142] proposed a model dependent on the stoichiometry of the protein complex comprising PCP. Other results suggest that the chain length determining the function of PCP depends on certain amino acid residues [143]. In the case of PCP involved in determination of the length of O-antigen chains in *P. aeruginosa*, it was reported that the protein level did not correlate with the length but with the amount of polysaccharide chains. The length of O-antigen chains was dependent on a specific amino acid residue in a coiled coil domain (coiled coils are specifically present in the periplasmic domains of PCP) [144,145] and evidenced that the length of the O-antigen chain was related to the stability of co-polymerase homodimerization with a positive correlation between dimer stability and production of longer chains. It was shown that single amino-acid mutations in PCP protein controlling the O-antigen chain length in *S. flexneri* causes subtle and localized structural changes that affect dramatically the chain-length distribution of LPS [146], and conserved glycine residues within the second transmembrane segment play a role in protein–protein interactions and contribute to the characteristic modality of O-antigen [147].

Polysaccharide co-polymerases proteins involved in O-antigen synthesis are subtly different from those involved in exopolysaccharide synthesis. The latter possess an additional extended cytoplasmic domain and are characterized by autophosphorylating kinase activity. This tyrosine kinase activity was shown to influence oligomerization of the protein, production of exopolysaccharide, and regulation of polymer chain lengths [148].

The most all-encompassing mechanism of PCP function is that proposed by Islam and Lam [98], called ‘chain-feedback-ruler’; it combines genetic and structural data about different PCPs involved in determining the lengths of exopolysaccharides and O-antigens in diverse bacteria. According to this model, polymerase and PCP proteins interact with each other and with the growing polysaccharide and keep it in a specified position, which promotes the addition of additional subunits. Extension of the polymer results in its folding into higher ordered structures, which causes weakening of the interactions between Wzy and PCP and termination of the ‘elongation’ of the polysaccharide [98].

The PssP protein was previously identified as having the characteristics of polysaccharide co-polymerases and being indispensable in the EPS synthesis in RtTA1 [64]. It is a large oligomeric protein located in the inner membrane [77]. It is essential for biosynthesis, as the deletion mutant does not produce EPS. Like other PCP proteins, PssP is involved in determining the length of chains produced due to the activity of polysaccharide polymerase PssT. Shortening of the protein at the C-terminus altered the ratio of HMW:LMW EPS produced by the mutant at the expense of the LMW fraction. This evidenced that the protein is not only indispensable for the EPS synthesis, but also necessary for the formation of the HMW form of EPS [64].

Exopolysaccharide transport outside the cell takes place through a channel formed in the Gram-negative outer membrane by oligomers of Wza proteins. Wza is a lipoprotein located in the outer membrane that forms an octameric α-helical channel spanning the membrane, but with the bulk of the protein existing as a large periplasmic structure [149,150,151,152]. An in vivo cross-linking strategy helped to trap the polysaccharide chain inside the Wza channel lumen, thus providing the first evidence that Wza is in fact a channel for export of a polymerized exopolysaccharide chain [153]. Wza and PCP proteins were shown to interact with each other forming a trans-envelope channel or platform opening into the cytoplasm and into the environment surrounding the cell, through which the polysaccharide is threaded outside the cell [154].

Polysaccharide co-polymerases were shown to form a trans-envelope tunnel for the growing polysaccharide chain by interacting with the outer membrane translocase [155]. The role of the outer membrane translocase in *R. leguminosarum* bv. *trifolii* is served by PssN. PssN was shown to be an outer membrane lipoprotein with an ability to form homooligomeric structures containing at least four protomers [106]. Its secondary structure content and topology characteristic for the Wza-like proteins, together with the recently revealed interactions with the PssP protein, gave a solid support for the hypothesis, that the protein serves as the outer membrane translocase for the exopolysaccharide (even though it was not possible to disrupt the gene) [77].

The mechanisms of biosynthesis and translocation of polysaccharides outside the cell must be temporally and spatially coordinated. This is only possible due to the interactions of the proteins involved in the processes. The existence of such a multiprotein complex has been proposed since the initial conception of the Wzx/Wzy-dependent synthesis pathway [156]. Research supports the existence of such multiprotein interactions in the Wzx/Wzy-dependent pathway, but in all cases, they represent fragmentary data in the various model bacteria. Several pieces of data concern formation of homooligomeric structures (with different numbers of protomers involved) by PCP [77,142,157,158,159,160], Wzy [77,132], and Wza proteins [106,152]. As initially expected, the number of data evidencing the existence of heterologous interactions is increasing. Marolda et al. [161] provided genetic evidence for the interactions between Wzx-Wzy andPCP, while other data concerned the interaction between Wzy and PCP [77,134,135,162,163], and PCP and Wza [77,155]. Recently, Nath and Morona [164] confirmed the long postulated physical interaction between the polymerase and the co-polymerase (Wzz in this case). They managed to co-purify Wzy and Wzz from membranes of *S. flexneri*. No interactions between glycosyltransferases acting in the Wzx/Wzy-dependent pathway have been experimentally demonstrated and still exist as a postulate.

**Figure 3:**
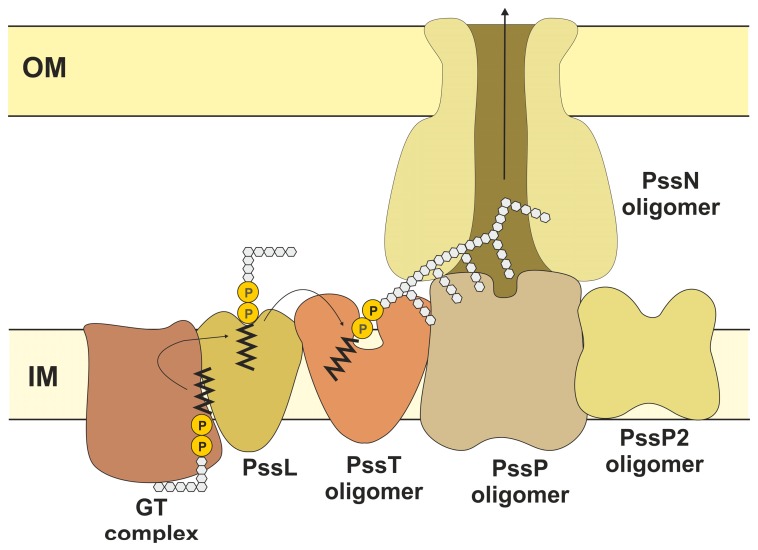
The proposed model of topology of the EPS transport system in *R. leguminosarum* bv. *trifolii*. The model is the result of a compilation of available data concerning the key proteins operating in the Wzx/Wzy-transport system and experimental data from the *R. leguminosarum* bv. *trifolii* model. Exopolysaccharide octasaccharide subunits are assembled on a lipid carrier by glycosyltransferases. Glycosyltransferases are shown as a single block, which presupposes the existence of a complex, although not apparent from the available experimental data. Exopolysaccharide octasaccharides are translocated to the outer leaflet of the IM (Inner Membrane) and PssL is proposed to serve this function. Subunits are then polymerized due to the concerted action of PssT polymerase and oligomeric PssP co-polymerase. Synthesis and transport proteins interact in a complex network, where the most prominent interactions involve: PssT-PssP and PssP-PssN. PssP2 protein is encoded within the Pss-II region, but was also shown to influence the length of EPS polymers and interact with PssP and PssT. OM: Outer membrane.

The studies on the architecture and mode of action of the EPS transport system in *R. leguminosarum* bv. *trifolii* provide some evidence for the existence of an essential and complex network of interactions between various components that are active at different stages of the EPS assembly process [77,106,163] (Figure 3). The oligomeric complex of PssP co-polymerase located in the inner membrane interacts with an oligomer formed by the outer membrane lipoprotein PssN and the oligomeric PssT polymerase [77]. It is proposed that co-polymerase PssP interacts also with PssL flippase, as indicated by the two-hybrid analysis results [77]. In light of the available data, PssP could constitute a bridge between the PssL flippase and the PssT polymerase and serve as a scaffold for the molecules of polymerase, according to the model proposed by Tocilj et al. [142]. The latter was reflected in the results of mapping of the interaction domains in PssT and PssP proteins. In the case of the PssP protein, it was shown that deletions of its different domains caused inability to form homooligomers, but did not completely abolish the protein’s ability to interact with PssT [77]. In line with this, mutants with shorter PssP variants produced EPS in which LMW fractions dominated [64]. In the case of the PssT protein, deletion of its C-terminal part made the protein more inclined to homooligomerization, but lack of the same domain made its interactions with PssP undetectable [77]. Deleting the C-terminal part of PssT in the mutant resulted in production of EPS with a majority of HMW chains [64] (Figure 3).

### 3.5. Other Genomic Regions that Contribute to (Exo)Polysaccharide Synthesis

Pss-I is not the only gene cluster involved in polysaccharide synthesis in *R. leguminosarum*; four other regions (designated Pss-II–Pss-V) with candidate genes were identified both in the chromosome and in the plasmid [88] (Figure 2). The Pss-II cluster, located approximately 200 kb from the Pss-I region, was almost identical in terms of the sequence and gene order with the corresponding part of the *Rlv* chromosome with only minor changes in comparison to *Rhe*. It was proposed that Pss-II could be engaged in LPS, EPS, or CPS biosynthesis, as it groups genes whose organization and sequence similarity indicate hypothetical functions of glycosyltransferases, Wzy-type polymerase, Wzx-type flippase, Wza-type translocase and PCP co-polymerase, i.e., key components of the Wzx/Wzy-dependent system [88,163] (Figure 2). Pss-III is an RtTA1 plasmid-borne polysaccharide biosynthesis region with an unknown function. A highly homologous segment was found in *Rlv* but not in the *Rhe* genome. Pss-IV and Pss-V are other chromosomal polysaccharide biosynthesis regions presumably functionally related with LPS biosynthesis. Both of these regions were syntenic to segments found in *Rlv* and *Rhe* (Figure 2). Moreover, many other putative LPS biosynthesis genes and small clusters (*acpXL*, *lpc*, *lpxQ*, *lpsB*, *kdtA*) were found to be dispersed in *R. leguminosarum* and *Rhe* genomes [87,165,166,167]. An apparent abundance of polysaccharide biosynthesis genes may be a demonstration of the metabolic redundancy typical for rhizobial genomes [87,89,168,169] or versatility of the rhizobial cell surface composed of a variety of polysaccharides, for which many adaptive biological functions have been attributed. Numerous Pss *loci* might encode alternate pathways for the synthesis of surface polysaccharides. For example, in *R. leguminosarum*, acidic CPS has a similar or even identical structure to the acidic EPS [51], differing only in noncarbohydrate residues [50]. Thus, genes in Pss-I, Pss-II, and Pss-III could contribute to CPS biosynthesis in *R. leguminosarum*. On the other hand, putative functional overlapping of polysaccharide biosynthesis genes located in distinct regions could be assumed and this assumption seems to be further supported by pleiotropic effects exerted by mutations in *pss* genes [170].

Recent functional studies carried out by Marczak et al. [163] provided important data related to the issue of potential functional interconnection/interchangeability of gene products encoded in distinct Pss clusters. PssP2 co-polymerase, encoded within the Pss-II, possesses most of the characteristic features of this family, e.g., specific topology. Tools enabling detection of remote homologues provided unambiguous annotation of *pssP2* as a PCP protein; however, less sensitive conventional sequence-based searches also pointed out the similarity with the Wzz/Wzc family [163]. The phenotype of the *pssP2* mutant, encoding a protein lacking 153 amino acids from its C-terminal cytoplasmic domain, indicated that the protein not only acted as a co-polymerase but its function was complementary to the PssP protein. The mutant produced more EPS than the wild type strain and with predominance of HMW fractions with chains with molecular masses higher than in the wild type [163].

One of the hypotheses regarding the regulation of the degree of polysaccharide polymerization assumes that glycosyltransferases must interact with a flippase and a co-polymerase and this is a step where regulation of the chain length could operate [171]. A key element of such a complex could be the priming glycosyltransferase PssA [119]. Interaction with the co-polymerase could control the flow of subunits from the tentative glycosyltransferase complex to the polymerization site. PssP2 was shown to interact with glycosyltransferase PssC and PssP/PssT proteins forming the ‘center of polymerization’ of EPS subunits [163] (Figure 3). Therefore, when PssP could serve as a bridge between the flippase and the polymerase and be involved in the synthesis of the HMW EPS fraction, PssP2 could be a link between the glycosyltransferases and the polymerase and be involved in the polymerization of the LMW EPS fraction. PssP2 and PssP may thus serve complementary/opposite roles in determining the extent of EPS polymerization [163]. The involvement of two similar proteins in EPS polymerization would resemble the involvement of the two Wzz proteins in the bimodal distribution of O-antigen in *S. flexneri*, where the two proteins compete to control the degree of polymerization [172]. Moreover, in *S. meliloti*, production of succinoglycan HMW and LMW forms requires more than one PCP paralogue [173]. The functions of other genes in the Pss-II region have not been studied; however, profound analysis thereof seems to be reasonable in order to clarify the functional importance of the abundance of homologues implicated in the synthesis of the same polysaccharide.

In *R. leguminosarum* and *Rhe*, a majority of polysaccharide biosynthesis genes are chromosomal with no distinguishable pExo plasmid such as that described previously for *S. meliloti* carrying *exo/exs* clusters [86]. Nevertheless, the plasmids do not appear to be entirely dispensable with respect to biosynthesis of surface structures. Some polysaccharide synthesis *loci* were mapped extrachromosomally in *Rlt*, *Rlv*, and *Rhe* [88,89,174]. RtTA1 derivatives cured of pRleTA1b or pRleTA1d and especially deleted in pRleTA1a (in which no genes related with polysaccharide biosynthesis were previously mapped) produced slightly higher amounts of EPS in comparison to the wild type, suggesting the existence of a complex regulatory network of EPS synthesis consisting of genes located on different plasmids and especially on pSym [175]. Extrachromosomal replicons were previously shown to confer the significant metabolic versatility to bacteria, which is important for their adaptation in the soil and nodulation competitiveness [7,8,176] and may also be exerted by modulation of the rhizobial surface composition. It has been shown that the quality and quantity of surface polysaccharides, particularly exopolysaccharides (EPSs) and lipopolysaccharides (LPSs), produced by rhizobia may influence both their autoaggregation and biofilm formation [177,178,179]. Rhizobial cell-to-surface interaction leading to the formation of biofilm plays a crucial role in root hair infection during symbiosis [180].

## 4. Conclusions

The prevalence and heterogeneity of bacterial surface polysaccharides as well as their diverse functions have long attracted the attention of scientists. This is particularly noticeable in studies of *Rhizobium*-plant symbiosis, where bacterial surface structures, especially exopolysaccharides (EPS), constitute a key determinant of the successful interaction between symbiotic partners. The comprehensive research of succinoglycan (EPS I) production in *S. meliloti* provided meaningful data about the role of EPS biosynthesis in a rhizobial model. However, further studies advanced our knowledge related to the complex pathway of EPSs export and function.

Firstly, the mechanisms in which EPSs determine beneficial interactions in legumes remained unknown for a long time, with a postulated signaling role of EPS, which was not recognized at the molecular level. Recent findings shed light on recognition of EPS by legume plants and the plant receptor protein EPR3 interacting with rhizobial EPS was identified. The kinase domain present in EPR3 transduces the signal from receptor to intracellular signaling cascades. Expression pattern of EPR3 suggests that EPS perception by receptor protein is important for the progression of nodule infection and development.

Secondly, it was previously concluded that repeating subunits of EPS are synthesized in cytoplasm, transported to the periplasm, polymerized, and then secreted outside the cell. The Wzx/Wzy-dependent pathway directing EPS synthesis was recognized in the *R. leguminosarum* model. It involves the action of the two key proteins: a Wzx-type flippase PssL and Wzy-type polymerase PssT. PssL flippase most probably transfers the EPS subunits from the cytoplasmic to the periplasmic leaflet of the inner membrane. PssT-dependent polymerization of undPP-linked oligosaccharide units takes place in the periplasm, with engagement of two periplasmic domains containing motifs with arginine residues important for the glycosyltransferase activity of the polymerase. PssP, a large oligomeric inner membrane protein, was identified as a polysaccharide co-polymerase involved in determining the length of EPS chains. PssN, an outer membrane homooligomeric lipoprotein, constitutes the outer membrane translocase allowing for the export of growing polysaccharide chains outside the cell. Evidence was provided that proteins constituting the Wzx/Wzy-dependent system of EPS assembly in *R. leguminosarum* bv. *trifolii* create a complex network wherein homooligomeric structures and heterocomplexes are formed.

Finally, the functional studies of putative *pss* loci, encoded outside the major Pss-I cluster of genes engaged in EPS production, revealed that abundance of polysaccharide biosynthesis genes may not only be a random trait and simply a demonstration of the metabolic redundancy typical for rhizobial genomes. PssP2 protein encoded within the Pss-II region in *R. leguminosarum* was shown to interact with proteins encoded within the Pss-I. It was hypothesized that similar co-polymerases PssP and PssP2 may play complementary/opposite roles in determining the polymerization degree of EPS. It was also demonstrated that *R. leguminosarum* derivatives cured of plasmids in which no *pss* genes were previously mapped produced slightly higher amounts of EPS in comparison to the wild-type strain. This suggested the existence of a complex network of interactions between the polysaccharide synthesis proteins, encoded in different compartment of rhizobial genomes. These latest findings also seem to draw the direction of future research in EPS biosynthesis in the rhizobial model. The open question is the function of numerous homologues genes implicated in the synthesis of the same polysaccharide and the interactions of their products with already recognized Pss proteins.
